# PredPSD: A Gradient Tree Boosting Approach for Single-Stranded and Double-Stranded DNA Binding Protein Prediction

**DOI:** 10.3390/molecules25010098

**Published:** 2019-12-26

**Authors:** Changgeng Tan, Tong Wang, Wenyi Yang, Lei Deng

**Affiliations:** 1 School of Computer Science and Engineering, Central South University, Changsha 410075, China; cgtan@csu.edu.cn (C.T.); tongwang@csu.edu.cn (T.W.); yangwenyi@csu.edu.cn (W.Y.); 2 School of Software, Xinjiang University, Urumqi 830008, China

**Keywords:** SSBs (single-stranded DNA-binding proteins), DSB (double-stranded DNA-binding proteins), protein sequence, gradient tree boosting, binding specificity

## Abstract

Interactions between proteins and DNAs play essential roles in many biological processes. DNA binding proteins can be classified into two categories. Double-stranded DNA-binding proteins (DSBs) bind to double-stranded DNA and are involved in a series of cell functions such as gene expression and regulation. Single-stranded DNA-binding proteins (SSBs) are necessary for DNA replication, recombination, and repair and are responsible for binding to the single-stranded DNA. Therefore, the effective classification of DNA-binding proteins is helpful for functional annotations of proteins. In this work, we propose PredPSD, a computational method based on sequence information that accurately predicts SSBs and DSBs. It introduces three novel feature extraction algorithms. In particular, we use the autocross-covariance (ACC) transformation to transform feature matrices into fixed-length vectors. Then, we put the optimal feature subset obtained by the minimal-redundancy-maximal-relevance criterion (mRMR) feature selection algorithm into the gradient tree boosting (GTB). In 10-fold cross-validation based on a benchmark dataset, PredPSD achieves promising performances with an AUC score of 0.956 and an accuracy of 0.912, which are better than those of existing methods. Moreover, our method has significantly improved the prediction accuracy in independent testing. The experimental results show that PredPSD can significantly recognize the binding specificity and differentiate DSBs and SSBs.

## 1. Introduction

Protein–DNA interaction is a crucial prerequisite for cell function, such as gene replication, transcription, and protein expression translation [1,2,3,4]. DNA can be categorized into single-stranded DNA (ssDNA) and double-stranded DNA (dsDNA). Accordingly, double-stranded DNA-binding proteins (DSBs) specifically bind with dsDNA, while single-stranded DNA-binding proteins (SSBs) specifically bind with ssDNA [5,6].

Knowledge about DNA-binding residues and binding specificity are important references for rational drug design [7,8,9,10]. The availability of binding specificity encourages researchers to focus on analyzing the specific binding sites of DSBs [11,12,13,14,15], the classification prediction of DNA-binding proteins [16,17,18], the function prediction of DNA-binding proteins [19,20,21,22] and the specificity of a protein to DNA binding [23,24], etc. However, the few existing methods for large-scale identification of DSBs and SSBs need further improvement. There are three main classification methods: (1) experimental techniques [25,26], (2) structure-based methods [27], and (3) sequence-based methods [28,29,30]. However, there are few large-scale identification methods for DSBs and SSBs.

In many earlier studies, biological functions have been mainly studied by X-ray crystallography, NMR, and filter binding assays [31,32,33]. However, the use of these experimental techniques for identifying DSBs and SSBs require expensive experimental setups and massive human resource allocations and are time-consuming. Hence, the development of computational methods has been emphasized by several investigators in the field. Initially, Wang et al. [27] proposed a support vector machine (SVM) method (Wang, 2014) with structure-based features related to surface clefts and OB-folds [30,34] as the input features. The results showed that this method achieved an accuracy of 0.8251 and an MCC of 0.6632. Because the gap between available sequences and structures of DNA binding proteins in UniProtKB/Swiss-Prot (www.uniprot.org) and the PDB (www.rcsb.org/pdb/) has been growing exponentially, structure-based methods can no longer meet the needs of high-throughput research [35,36]. Subsequently, Wei Wang et al. [37] developed a machine learning method (Wang, 2017) with only single sequence information such as overall amino acid composition (OAAC) features, dipeptide compositions, and position-specific scoring matrix profiles (PSSMs). The results showed an accuracy of 88.7% and an AUC (area under the curve) of 0.919 on the benchmark datasets.

Although these computational prediction methods have been gradually developed, there are still some problems that make DSB and SSB classification prediction a very challenging task. On the one hand, the performance of commonly used feature extraction methods is still unsatisfactory, and the sequence information cannot be fully utilized to extract more effective features. On the other hand, novel feature selection algorithms and high-performance ensemble learning algorithms, such as gradient tree boosting (GTB), are rarely used in this field.

**Figure 1:**
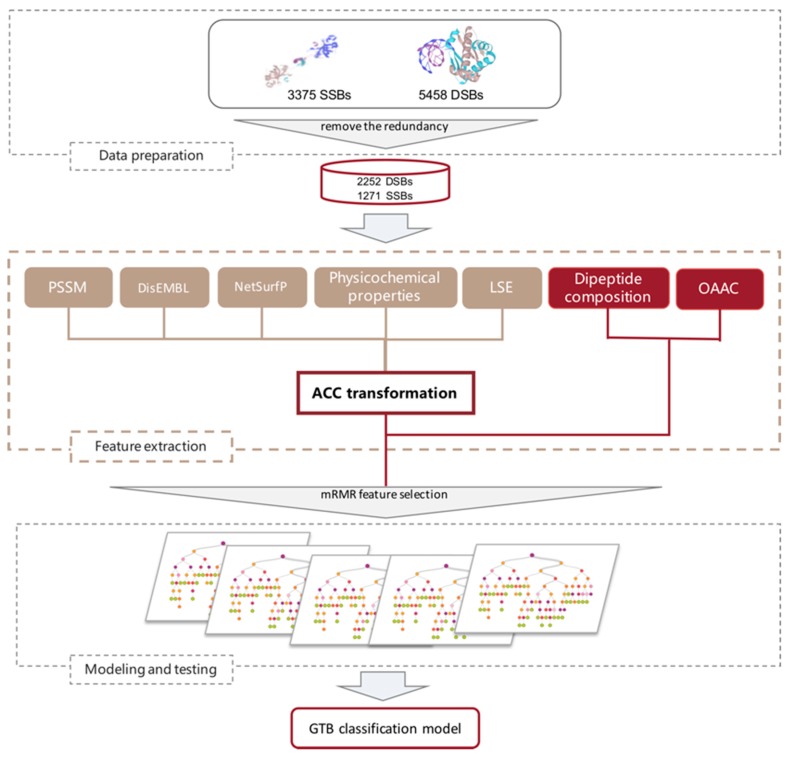
Workflow of PredPSD.

In this work, we have developed a novel approach, PredPSD, for classifying DSBs and SSBs through a more complete combination of sequence features, such as local structural entropy (LSE), NetSurfP, and DisEMBL. The results show an accuracy of 91.2% and an AUC (area under the curve) of 0.956 on benchmark datasets and indicate that the GTB algorithm and novel feature combinations are essential determinants in the classification of DSBs and SSBs. Furthermore, our algorithm achieves a significantly improved overall performance on an independent dataset. The workflow of our method is shown in Figure 1.

## 2. Results

### 2.1. Analysis of mRMR Results

**Figure 2:**
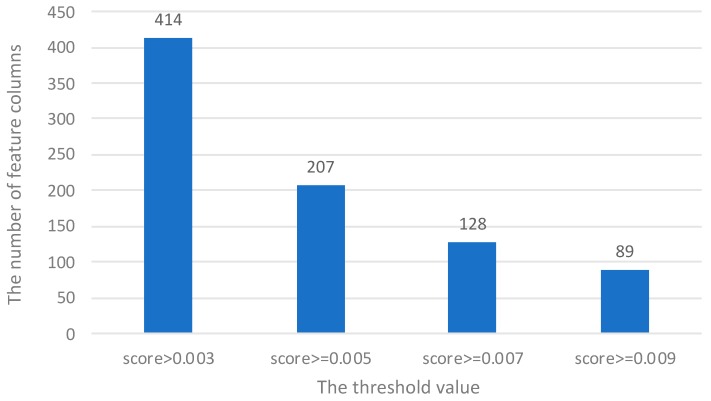
Taking the maximum correlation score as a threshold, the number of feature columns changes with the threshold value.

We calculated a total of 1510 sequence features for each protein, including local structural entropy (LSE), NetSurfP, DisEMBL, overall amino acid composition (OAAC), dipeptide composition, PSSM profiles, and physicochemical properties. The maximum correlation between feature and category is computed. Different subsets of characteristics are obtained by setting different thresholds. The number of features changes with the threshold value, as shown in Figure 2.

Through the verification of the machine learning algorithm in terms of time complexity and accuracy, we finally set the threshold as 0.005. Therefore, we obtained an optimal set of 207 features. These selected features are shown in Supplementary Table S1. We also compared the experimental performance before and after using the feature selection algorithm. Specific experimental performance comparison results are shown in Supplementary Table S2 and S3.

**Figure 3:**
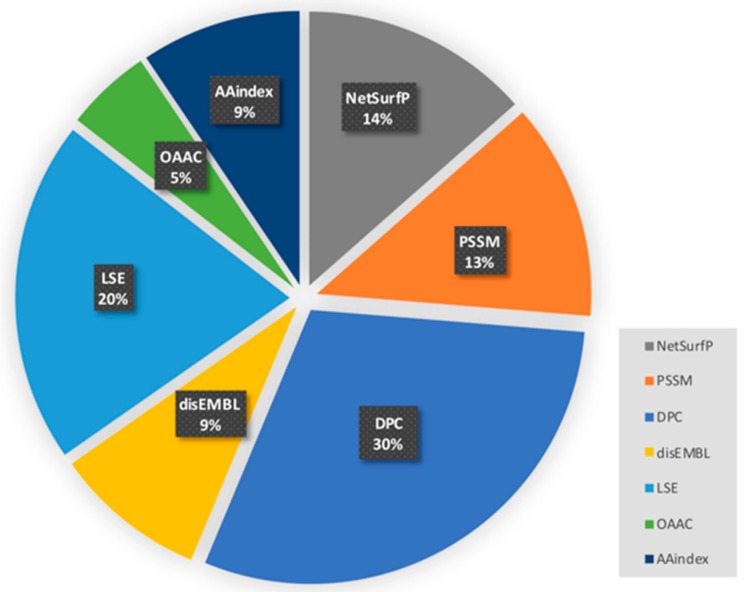
Probability of each feature being selected.

To evaluate the mRMR method, we calculated the ratio of the number of selected features to the number of candidate features. A pie chart of the selected feature extraction methods and the corresponding ratios is illustrated in Figure 3, which shows that the total probability of the selected features obtained by NetSurfP, LSE, and DisEMBL is 43%. As expected, the introduction of sequence-based features has a strong correlation between the classification of two different types of proteins.

**Figure 4:**
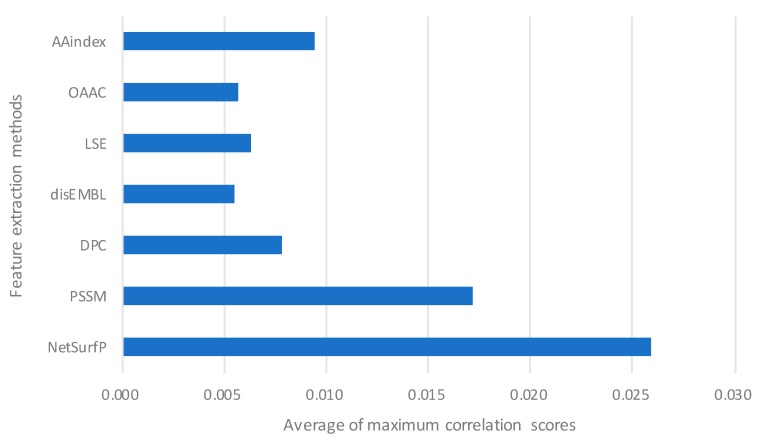
The top five maximum correlation scores of features obtained by different feature extraction methods were calculated.

Through the analysis of 207 optimal features, we found that most of the top 10 selected features are obtained by NetSurfP and PSSM. Among the 207 features, the cumulative score of dipeptide composition features was close to that of PSSM features, ranking third. This shows the importance of global sequence information. The average score of NetSurfP was much higher than other features, suggesting that the binding specificity of single-stranded and double-stranded DNA-binding proteins is closely correlated with structural information. The average value of the maximum correlation scores of each feature is shown in Figure 4.

### 2.2. Feature Extraction Results Analysis

**Figure 5:**
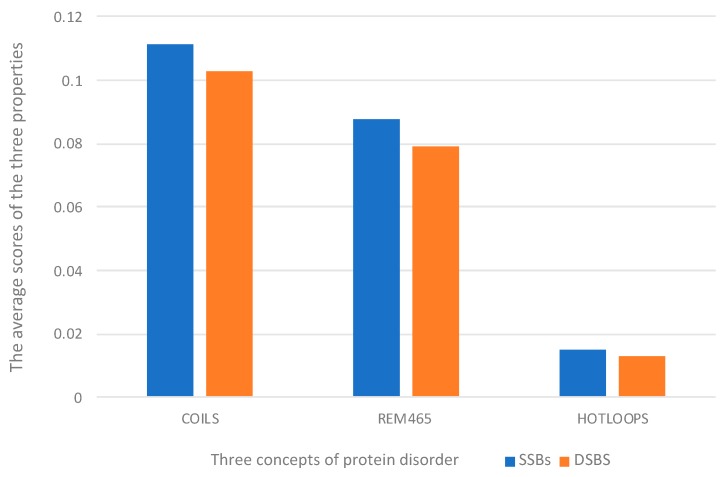
Average scores of DisEMBL’s three properties on SSBs and DSBs.

DisEMBL is a method for predicting disordered regions in protein sequences with high accuracy. It profits from predicting protein disorder according to multiple definitions, including COILS, REM465, and HOTLOOPS [38]. To observe the prediction of the above three concepts more intuitively, we used a histogram to compare the prediction results processed by ACC algorithm, as shown in Figure 5. By observing the chart, we can easily find that the average scores of the three properties on SSBs are slightly higher than those on DSBs. This specificity is what we use to distinguish the two proteins.

In addition, the feature matrix of each protein obtained by NetSurfP contains five properties in total, and 30-dimensional row vectors are obtained after five iterations of ACC transformation. Overall, it can be concluded that the scores of three of the properties of DSBs (relative surface accessibility (RSA), absolute surface accessibility, and probability for beta-strand) are significantly higher than those of SSBs. Although SSBs and DSBs have many similar properties, proteins can specifically recognize single and double-stranded DNA and bind to the right place. The essential reason may be related to their structural characteristics. Therefore, NetSurfP plays a crucial role in the prediction.

### 2.3. Predictive Performance of Features

**Figure 6:**
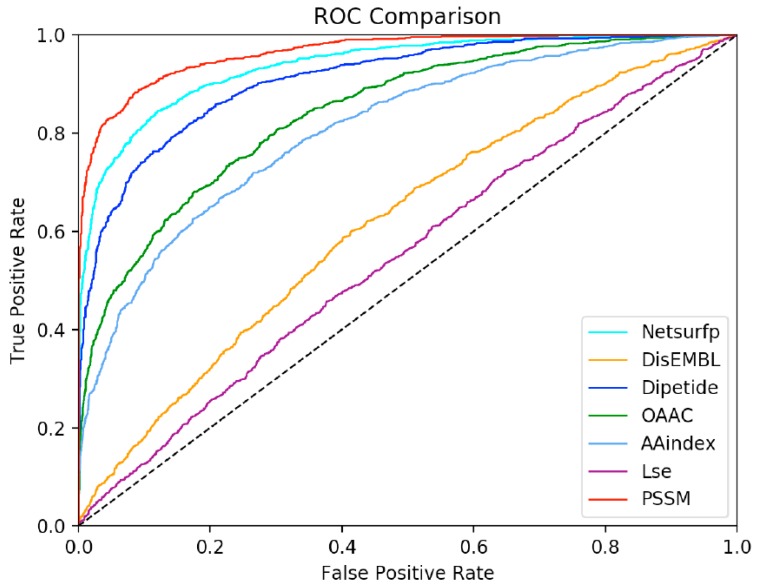
ROC curves of the GTB model on the training set. Different colors represent different types of features.

To further verify the predictive performance of the seven features, we constructed a classification model for each feature by combining the GTB algorithm with 10-fold cross-validation. Figure 6 depicts the ROC curves for the different features.

Different features have different abilities to identify SSBs and DSBs correctly. Consistent with the analysis of the feature selection results, PSSM had the optimal predictive ability for positive and negative samples, followed by NetSurfP. Overall, each feature plays an indispensable role in the prediction. Because of the low dimension of the feature matrix of features AAindex, LSE, and DisEMBL, they are not individually outstanding. However, they have functional complementarity and thus can play better roles when combined. For performance evaluation, we used several widely used measurement methods: accuracy, sensitivity (SN), specificity (SP), F1-score (F1), Matthew’s correlation coefficient (MCC), and area under ROC curve (AUC). Table 1 shows that the three features with the highest accuracies are PSSM, NetSurfP, and dipeptide, reaching 0.913, 0.874, and 0.836, respectively. On the one hand, it shows that the feature extraction algorithm we selected is very effective. On the other hand, it also reflects the fit between the GTB algorithm and this study.

### 2.4. Comparison with Previous Work

To our knowledge, there is only one existing study on SSB and DSB prediction based on sequence information, which uses two machine learning algorithms, SVM (Wang 2017_SVM) and RF (Wang 2017_RF). Furthermore, the accuracy of the two methods trains the model on Uniprot1065 dataset with an accuracy of 0.860 and 0.887, respectively. Finally, due to the defects of high sensitivity and low specificity in the performance of the model based on random forest, the SVM model was taken as the optimal model. In this paper, based on our larger dataset, we compare PredPSD with the above two methods. Overall, our method is superior to the existing methods in terms of accuracy, specificity, and sensitivity.

**Figure 7:**
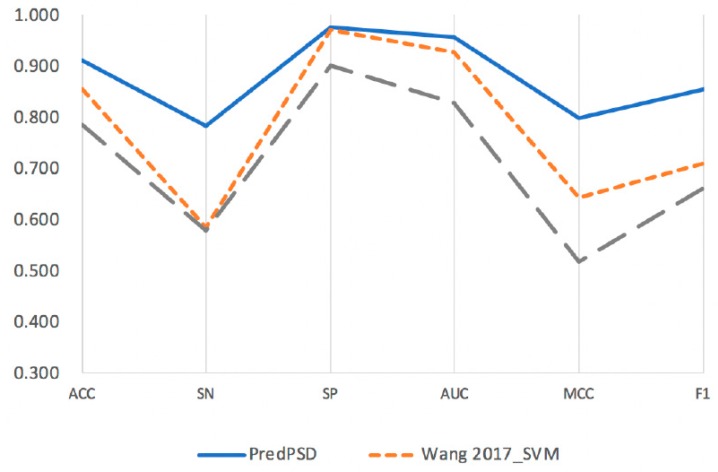
Performance comparison between our method and three existing classification methods on SSBs and DSBs from our training set. The existing classification methods are Wang 2017_SVM (based on sequence information and an SVM algorithm) and Wang 2017_RF (based on sequence information and an RF algorithm), all proposed by Wang W. et al. To our knowledge only these two methods are all relevant to this study.

The results are presented in Figure 7 and Table 2. In general, we can see that our PredPSD method shows an advantage in all six metrics (Accuracy, SN, SP, F1, MCC, and AUC) on the training set. The optimal predictive energy of our method in the training dataset after redundancy removal is as follows: accuracy of 0.912, SN of 0.784, SP of 0.975, AUC of 0.956, and MCC of 0.799. By comparing with Wang 2017_SVM, we can see that the F1 score of our PredPSD approach has been significantly improved by 14.5%.

### 2.5. Comparative Analysis of Independent Test Results

**Figure 8:**
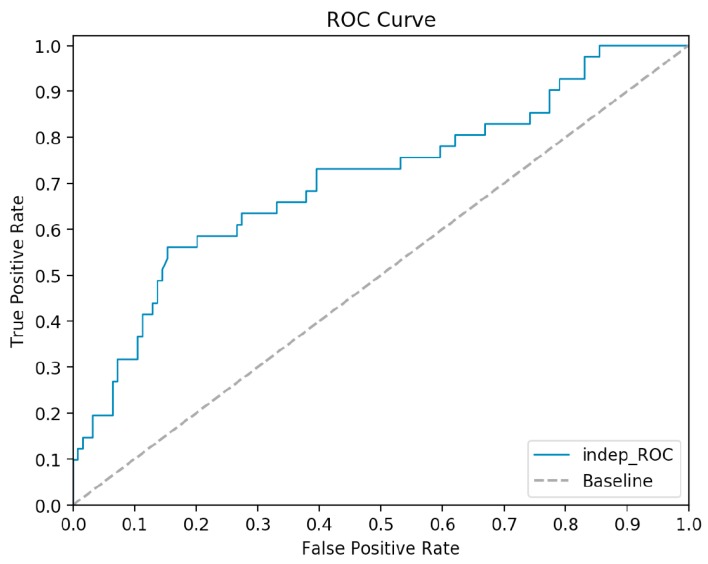
ROC curve based on the independent dataset.

We used the trained model to complete the test on the independent dataset to further verify the generalization ability of the model and avoid overfitting. The processing of the independent dataset is consistent with that of the training set. The exception is that no additional feature selection is carried out for the independent dataset, and the feature matrix of the independent dataset is reconstructed directly using the feature selection results obtained from the training set. The experimental results show that PredPSD presents the best predictability based on the combination of all features: accuracy of 0.770, SN of 0.512, SP of 0.855, AUC of 0.708, and F1 of 0.525. The ROC curve is shown in Figure 8.

The information in the figure indicates that PredPSD is superior to the existing optimal method of predicting SSBs and DSBs based on sequence information in the independent dataset. Detailed results of the comparisons between our approach and existing methods are shown in Table 3. From the overall analysis of the method comparison table, it can be seen that PredPSD has the most prominent comprehensive performance among the three methods. First, compared with method Wang 2017_SVM, our accuracy improved by 6.7%, and other evaluation indexes are greatly improved. The specificity and AUC values of the two methods are similar, but the sensitivity, MCC, and F1 are all improved by more than 10%. In particular, the MCC value increased by 18.8%. Second, although the specificity of Wang 2017_RF method is less than 0.05 different from our method, the sensitivity is only 0.341, so it has low validity. Finally, by comparing the performance of the three algorithms on the training set and the independent dataset, the results show that our model has stronger generalization ability. Furthermore, we annotate all the SSBs we used for classification by using the InterProScan tool. Then, proteins belonging to OB folds, KH domains, RRMs, and whirly domains were obtained by processing annotation information. Finally, the prediction results show that PredPSD has higher recognition ability for proteins with OB folds and whirly domains. However, the prediction accuracy of proteins with KH domains and RRMs structures is low. The reason may be that KH domain and RRMs can also specifically bind ssDNA sequences specifically, but these domains generally have a smaller ligand-binding site than OB folds and thus specify for fewer positions [39].

## 3. Materials and Methods

### 3.1. Datasets

For sequence-based feature calculation, we extracted 8833 DNA-binding proteins. Which contains 2136 DSBs and 339 SSBs obtained from the literature of Wang et al. [37] And the other part is collected from UniProtKB/Swiss-Prot (www.uniprot.org). To eliminate redundancy, CD-HIT was used to remove proteins with a sequence similarity > 70% [40]. Finally, we obtained a dataset of DNA binding proteins containing 1271 SSBs and 2252 DSBs. We took SSBs as the positive sample dataset and DSBs as the negative sample dataset.

Furthermore, to evaluate the classification performance and avoid overfitting, we obtained a non-redundant independent set of 124 DSBs and 41 SSBs from the PDB (www.rcsb.org/pdb/), which has the following characteristics: (1) sequence similarity with the training set is less than 40%; (2) sequence length is greater than 40 residues; (3) structure of each DNA-binding protein is known, and the resolution is better than 3 Å.

### 3.2. Feature Extraction

Selecting representative features is a crucial step because they directly determine prediction performance [41,42]. Seven sequence-based feature extraction methods were used: local structural entropy (LSE), NetSurfP, DisEMBL, overall amino acid composition (OAAC), dipeptide composition, PSSM profiles, and physicochemical properties. These methods have proven to be associated with the classification of DSBs and SSBs or have been used in similar fields. A more detailed description of how to extract and encode these different sequence-based features is provided below.

#### 3.2.1. Local Structural Entropy (LSE)

LSE describes the conformational isomeric degree sequence of small proteins [43,44], which can provide useful information for protein classification. We used a method of computing LSE directly from sequence information.

#### 3.2.2. NetSurfP

NetSurfP [45] is a tool that has been used to predict the secondary structure and surface accessibility of proteins based on sequence information [46]. It is an architecture composed of neural network [47] training on proteins with known structures. Local structural features such as relative surface accessibility, probability for alpha-helix, and probability for beta-strand play important roles in revealing the function of proteins and can also be used for protein classification.

#### 3.2.3. DisEMBL

DisEMBL [38] is a method for predicting disordered regions in protein sequences. Disordered regions of proteins usually contain short linear peptide motifs such as SH3 ligands and targeting signals that are important for the classification of proteins by function.

#### 3.2.4. Overall Amino Acid Composition (OAAC)

Existing studies have shown that the overall amino acid composition of 20 standard amino acids is a sequence feature widely used in the field of protein identification. Using the OAAC method, a 20-dimensional vector can be calculated, where each value describes the frequency of the amino acid in the sequence. Previous literature has shown that the square root of probability is more conducive to research [48]. Therefore, the following formula is used to define the probability

(1) $$p_{i}=\sqrt{\frac{n_{i}}{L}}\;\left(i=1,2,\ldots 20\right)$$

where $n_{i}$ refers to the number of times the ith amino acid in the protein sequence. Given a protein sequence S with length *L*, its OAAC feature vector can be expressed as

(2) $$S_{OAAC}=\left[p_{1},p_{2},p_{3},\cdots ,p_{20}\right]$$

#### 3.2.5. Dipeptide Composition

A dipeptide is a compound formed by the dehydration and condensation of two amino acids. Here, it can be viewed as any combination of two amino acids. Since there are 20 kinds of amino acids, a total of 400 dipeptide compositions are possible [49,50]. Dipeptide composition is obtained by calculating the ratio of the number of occurrences of dipeptides in the sequence to the sequence length. Dipeptides are spaced differently in protein sequences. In this paper, three common distributions (0, 1, and 2) were selected [51]. Eventually, each protein will generate a vector of 1,200 dimensions. Dipeptides probabilities are defined as

(3) $$p_{ab}\left[i\right]=\frac{D_{ab}\left[i\right]}{N-1}\;\left(a,b=G,R,L\cdots ;\;i=0,1,2\right)$$

where $D_{ab}\left[i\right]$ represents the number of dipeptides formed by two amino acids a and *b* at an interval of i , and *N* is the length of the protein sequence.

#### 3.2.6. PSSM

In this work, the practical significance of the position-specific scoring matrix (PSSM) is to find the conserved features of specific conserved positions from the sequences of DSBs and SSBs that can be used for the classification of the two types of proteins [52]. The PSSM of the residues is implemented by the PSI-BLAST [53] program, which contains essential evolution information through three iterations. A 20-dimensional vector with integer values represents each residue. These values represent the frequency of mutations at various locations in the sequence, and the PSSM can be expressed as

(4) $$PSSM_{S}=\left[\begin{array}{ccc} G_{1,1} & \cdots & G_{1,20}\\ \vdots & \ddots & \vdots \\ G_{L,1} & \cdots & G_{L,20} \end{array}\right]$$

where $PSSM_{S}$ represents a 20×L matrix of protein S , and L represents the length of the protein sequence. *G_i,j_* is the probability score of the amino acid at position i of the S protein sequence being replaced by the basic amino acid encoding j during evolution.

#### 3.2.7. Physicochemical Properties

The physicochemical properties of proteins are intuitive and straightforward basic characteristics with reliable physical and biological meanings [54,55]. We selected 28 typical numerical properties [56] commonly used for DNA binding protein classification in the database AAindex [57] to encode amino acids. A protein sequence of length L can be expressed as a matrix of 28 × *L* dimensions, where each row represents the attribute value of the residue at that location. The list of AAindex physicochemical properties we used can be found in Supplementary Table S4.

### 3.3. Feature Transformation

Protein sequences usually have different lengths. However, machine learning-based methods such as GTB require fixed-length vectors for training. Here, we introduce the autocross-covariance (ACC) transformation to transform protein sequences into fixed-length vectors by measuring the correlation of two properties along the protein sequence [58]. The ACC method contains two variables, AC and CC. AC is used to calculate the correlation of two residues with a distance of lg in the same attribute. It is defined as

(5) $$AC\left(i,lg\right)=\overset{L-lg}{\underset{j=1}{\sum }}(s_{ij}-\bar{s_{i}})(s_{ij+lg}-\bar{s_{i}})\;/\;\left(L-\mathrm{lg}\right)$$

where i is one of the columns corresponding to a residue, lg is the distance between the two residues, L is the number of residues in the protein sequence, $s_{ij}$ is the value of the ith row and the jth column in the matrix, and $\bar{s_{i}}$ is the average score for L columns

(6) $$\bar{s_{i}}=\overset{L}{\underset{j=1}{\sum }}s_{ij}/L$$

Therefore, the number of AC variables is obtained by multiplying the number of attributes by the number of LG values. LG is the maximum of the intervals lg.

The CC variable calculates the relationship between two different attributes. The specific calculation is

(7) $$\mathrm{CC}\left(i1,i2,lg\right)=\overset{L-lg}{\underset{j=1}{\sum }}\left(S_{i1,j}-\bar{S_{i1}}\right)\left(S_{i2,j+lg}-\bar{S_{i2}}\right)/\left(L-lg\right)$$

where i1 and i2 represent the columns corresponding to two different attributes, and $\bar{S_{i1}}$ ($\bar{S_{i2}}\;$) is the average value of the ith column. We finally choose the ACC variable, which is the result of the combination of the AC variable and the CC variable.

**Figure 9:**
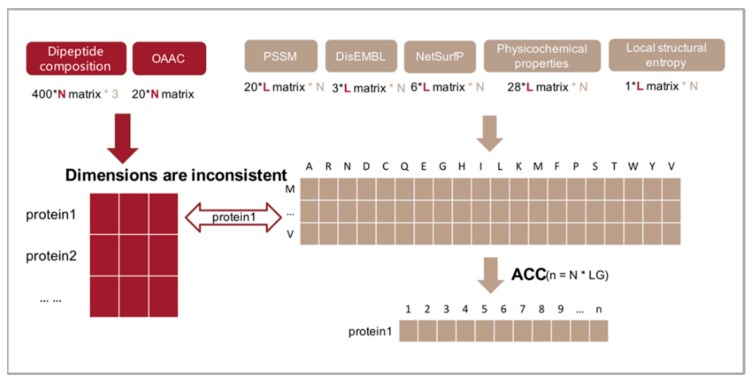
Application of the ACC transformation in this study. For example, protein1 refers to the name of a protein, and features extracted based on its sequence information are vectors, while others are matrices. In this case, the matrix can be converted to a vector.

In this work, ACC transformation was used on the matrix obtained by physicochemical, PSSM, DisEMBL, and NetSurfP. The feature matrix of each protein is converted into a vector, where the parameter LG is set to 5, and the visual description is shown in Figure 9.

### 3.4. Feature Selection

A feature selection algorithm can help us understand the characteristics of features, and it plays a vital role in further optimizing the algorithm and improving classification accuracy [59]. The candidate feature space selected by minimal-redundancy-maximal-relevance criterion (mRMR) is more representative [60,61]. Therefore, based on the candidate features of 1520 columns, we further select the optimal feature subset using the mRMR algorithm. The general process of mRMR feature selection is as follows: first, the data are processed and stored with the appropriate data structure. Second, the distribution and mutual information between features and between features and response variables are calculated. Finally, the features are scored and sorted by mRMR. A score file maximum correlation and minimum redundancy for each column feature are then obtained.

Specifically, the correlation between feature subset and category is calculated by the mean of the information gain of each feature and category

(8) $$\max D\left(S,c\right),\;D=\frac{1}{\left|S\right|}\underset{x_{i}\in S}{\sum }I\left(x_{i};c\right)$$

where S is a feature set, c is the target category, $x_{i}$ represents a feature in the feature set S, and $I\left(x_{i};c\right)$ indicates all the mutual information values between a single feature $x_{i}$ and class c. maxD(S,c) represents that $x_{i}$ in S has the highest dependence on the target class c.

The redundancy between two features is calculated by the sum of mutual information between two features and then divided by the square of the feature number in the subset

(9) $$\min R\left(S\right),\;R=\frac{1}{{\left|S\right|}^{2}}\underset{x_{i},x_{j}\in S}{\sum }I\left(x_{i};x_{j}\right)$$

where $I\left(x_{i};x_{j}\right)$ is the mutual information between two classes. If the two classes are highly dependent on each other, removing one of them will not affect classification performance.

### 3.5. Classification Model and Performance Evaluation

Gradient tree boosting (GTB) [62] is an integrated base classifier decision tree algorithm that can be used for classification and regression problems [63,64,65,66,67]. In this study, it is assumed that SSBs and DSBs belong to a binary classification problem. We finally chose the gradient tree boosting of ‘sklearn.ensemble’ as the classification method, because it can better address mixed types of data and is more robust to outliers. GTB produces a decision tree composed of J leaf nodes by reducing the gradient direction of each sample point and its residuals [68,69,70]. In the experiment, the optimal parameters of GTB were selected by 10-fold cross-validation on the benchmark dataset using a grid search strategy. These performance evaluations we use are defined as

(10) SN=TP/(TP+FN)

(11) SP=TN/(TN+FP)

(12) $$F1=\frac{2\times \mathrm{Recall}\times \mathrm{Precision}}{\mathrm{Recall}+\mathrm{Precision}}$$

(13) $$\mathrm{Accuracy}=\frac{\mathrm{TP}+\mathrm{TN}}{\mathrm{TP}+\mathrm{TN}+\mathrm{FP}+\mathrm{FN}}$$

(14) $$\mathrm{MCC}=\frac{\mathrm{TP}\times \mathrm{TN}-\mathrm{FP}\times \mathrm{FN}}{\sqrt{\left(\mathrm{TP}+\mathrm{FP}\right)\left(\mathrm{TP}+\mathrm{FN}\right)\left(\mathrm{TN}+\mathrm{FP}\right)\left(\mathrm{TN}+\mathrm{FN}\right)}}\;$$

In these equations, TP (the number of SSBs correctly classified), TN (the number of DSBs correctly classified), FP (the number of DSBs that are misclassified as SSBs) and FN (the number of SSBs that are misclassified as DSBs) represent true positives, true negatives, false positives and false negatives, respectively. Here, the category of SSBs is called a positive class, and the category of DSBs is called a negative class. Among these evaluation indexes, MCC and F1 reflect the overall performance of the classifier and can better evaluate the performance of the classifier in the case of unbalanced data. The classification performance was evaluated by 10-fold cross-validation.

## 4. Conclusions

In this work, we have proposed a novel method PredPSD for the classification prediction of SSBs and DSBs. The method is based on the gradient tree boosting (GTB) algorithm [71], and the model was trained on 1271 SSBs and 2252 DSBs non-redundant datasets. In the course of the experiment, we introduced three feature extraction algorithms that have never been used in this research problem that can extract a variety of features derived from multiple sequences. At the same time, we also combined four commonly used algorithms. We comprehensively evaluated the effects of different sequence extraction methods on prediction performance. Then, we used the ACC transformation algorithm to solve the problem of inconsistent feature dimensions. The mRMR method was used to obtain a set of optimal features with maximum correlation and minimum redundancy, and the strong specificity of the new features to distinguish different types of proteins was verified.

PredPSD resulted in prediction accuracy of 91.2% and an AUC of 0.956 on the training set through 10-fold cross-validation. Furthermore, in the independent dataset, PredPSD can achieve an accuracy of 77.0% and an SP of 0.855. The results show that the prediction performance of PredPSD is better than that of previous methods. Our study provides a complementary and effective method to predict SSBs and DSBs more accurately.

## Figures and Tables

**Table 1:** Performance of classification models derived from different types of features

Features	Accuracy	SN	SP	AUC	MCC	F1
PSSM	0.913	0.814	0.968	0.968	0.809	0.870
AAindex	0.759	0.514	0.899	0.810	0.457	0.604
OAAC	0.778	0.572	0.895	0.843	0.503	0.649
NetSurfP	0.874	0.780	0.927	0.938	0.723	0.817
LSE	0.668	0.430	0.782	0.646	0.219	0.456
Dipeptide	0.838	0.645	0.948	0.912	0.644	0.741
DisEMBL	0.682	0.421	0.818	0.670	0.258	0.477

**Table 2:** Performance of all feature descriptors with GTB algorithms based on our training set and comparisons with existing methods

Features	Accuracy	SN	SP	AUC	MCC	F1
PredPSD	0.912	0.784	0.975	0.956	0.799	0.854
Wang 2017_SVM	0.855	0.585	0.972	0.927	0.643	0.709
Wang 2017_RF	0.784	0.578	0.902	0.827	0.518	0.661

**Table 3:** Performance of all feature descriptors with GTB algorithms based on the independent dataset and comparisons with existing methods.

Features	Accuracy	SN	SP	AUC	MCC	F1
PredPSD	0.770	0.512	0.855	0.708	0.373	0.525
Wang 2017_SVM	0.703	0.366	0.814	0.692	0.185	0.380
Wang 2017_RF	0.721	0.341	0.847	0.620	0.203	0.378

## Supplementary Materials

The following are available online at https://www.mdpi.com/1420-3049/25/1/98/s1.
