# Supplementary material for: PredPSD: A Gradient Tree Boosting Approach for Single-Stranded and Double-Stranded DNA Binding Protein Prediction
Source: Molecules. 2019 Dec 26;25(1):98. doi: 10.3390/molecules25010098 (PMC6982935; doi:10.3390/molecules25010098)
Supplement: Supplementary file 1 [file molecules-25-00098-s001.pdf]

Supplementary Material

PredPSD: A Gradient Tree Boosting Approach for Single-Stranded and Double-Stranded DNA Binding Protein Prediction

Changgeng Tan [1,†], Tong Wang [1,†], Wenyi Yang [1], and Lei Deng [1,2,*]

1. School of Computer Science and Engineering, Central South University, Changsha, 410075, China
2. School of Software, Xinjiang University, Urumqi 830008, China; cgtan@csu.edu.cn (C.T.); tongwang@csu.edu.cn (T.W.); yangwenyi@csu.edu.cn (W.Y.)

† These authors contributed equally to this work.

* Correspondence: leideng@csu.edu.cn; Tel.: +86-731-82539736 (L.D.)

1 Supplementary Tables

The optimal feature subset obtained by mRMR method is distributed in the feature matrix obtained by seven feature extraction algorithms, including local structure entropy (LSE), NetSurfP, DisEMBL, total amino acid composition (OAAC), dipeptide composition, PSSM spectrum, and physicochemical properties. These selected features are shown in Supplementary Table S1.

Supplementary Table S1. Column numbers of 207 features obtained by the mRMR algorithm

Feature	The selected column Numbers
**DisEMBL**	0, 1, 4, 13
**AAindex**	1,2,10,11,25,29,30,32,36,45,46,57,58,59,62,68,69,70,73,74,75,82,84,86,87,90,92,94,95,97,100,101,102,103,104,107,108,109,109,111
**OAAC**	3,9,14
**NetSurfP**	0,1,3,4,6,7,9,10,16,22,27,28
**LSE**	1,2,3
**Dipeptide**	37,47,54,63,74,92,107,120,126,142,145,153,154,162,176,182,183,193,195,198,199,209,220,227,254,261,267,269,289,292,297,299,328,343,367,400,413,425,428,433,434,438,440,443,445,459,463,466,467,476,479,493,501,514,530,542,545,549,551,554,557,568,574,587,590,619,633,634,638,663,671,674,685,697,707,773,774,775,790,793,806,843,847,878,928,941,942,945,952,955,966,967,974,987,1003,1018,1033,1066,1084,1103,1180,1182,1183,1185,1189,1193,1194,
**PSSM**	2,4,5,6,9,10,12,16,17,18,19,22,23,24,25,26,28,29,31,33,36,37,45,47,58,59,60,63,67,69,77,78,84,92,94,95,96,98

Supplementary Table S2. List of AAindex physicochemical properties

AAindex	AAindex	AAindex	AAindex
CHOP780202	CIDH920103	CIDH920105	FAUJ880109
GEIM800106	KANM800102	KLEP840101	KRIW710101

PALJ810107	QIAN880123	RACS770103	RADA880108
ZIMJ680104	AURR980120	MUNV940103	NADH010104
FAUJ880111	FINA910104	GEIM800104	NADH010106
LIFS790101	MEEJ800101	OOBM770102	GUYH850105
ROSM880102	SWER830101	ZIMJ680102	MIYS990104

The mRMR feature selection method was finally determined through the following comparative experiment: (1) We evaluate the performance with 10-fold cross-validation on the feature-reduced dataset and the full-featured dataset. The result shows that the feature selection algorithm is useful in performance and time cost. (2) We use the recursive feature elimination with cross validation (RFECV) method (GTB-based) to select the top 134 features as an optimal feature set for classification. Then, we also use the mRMR feature selection method for comparative experiments.

Supplementary Table S3. Prediction performance of GTB-based RFECV algorithm in comparison with mRMR feature selection method on training dataset

Method	Accuracy	SN	SP	AUC	MCC	F1
**Full-featured**	0.887	0.748	0.966	0.955	0.755	0.826
**RFECV**	0.917	0.819	0.970	0.973	0.817	0.874
**mRMR**	0.912	0.784	0.975	0.956	0.799	0.854

Supplementary Table S4. Prediction performance of GTB-based RFECV algorithm in comparison with mRMR feature selection method on independent dataset

Method	Accuracy	SN	SP	AUC	MCC	F1
**Full-featured**	0.703	0.415	0.798	0.671	0.211	0.410
**RFECV**	0.715	0.415	0.815	0.687	0.231	0.420
**mRMR**	0.770	0.512	0.855	0.708	0.373	0.525

According to the results in Supplementary Table S3, the prediction performance based on RFECV is better than that based on mRMR on the training set. Supplementary Table S4 shows that the model trained based on mRMR has better performance on the independent dataset. Also, because we have 1510 features, the RFECV method takes much longer than method mRMR.
